# The impact of regional heterogeneity in whole-brain dynamics in the presence of oscillations

**DOI:** 10.1162/netn_a_00299

**Published:** 2023-06-30

**Authors:** Yonatan Sanz Perl, Gorka Zamora-Lopez, Ernest Montbrió, Martí Monge-Asensio, Jakub Vohryzek, Sol Fittipaldi, Cecilia González Campo, Sebastián Moguilner, Agustín Ibañez, Enzo Tagliazucchi, B. T. Thomas Yeo, Morten L. Kringelbach, Gustavo Deco

**Affiliations:** Department of Physics, University of Buenos Aires, Buenos Aires, Argentina; National Scientific and Technical Research Council (CONICET), CABA, Buenos Aires, Argentina; Cognitive Neuroscience Center (CNC), Universidad de San Andrés, Buenos Aires, Argentina; Center for Brain and Cognition, Computational Neuroscience Group, Universitat Pompeu Fabra, Barcelona, Spain; Neuronal Dynamics Group, Department of Information and Communication Technologies, Universitat Pompeu Fabra, Barcelona, Spain; Global Brain Health Institute, University of California, San Francisco, CA, USA; and Trinity College Dublin, Dublin, Ireland; Latin American Brain Health Institute (BrainLat), Universidad Adolfo Ibáñez, Santiago, Chile; Trinity College Institute of Neuroscience (TCIN), Trinity College Dublin, Dublin, Ireland; Centre for Sleep and Cognition, Centre for Translational MR Research, Department of Electrical and Computer Engineering, N.1 Institute for Health and Institute for Digital Medicine, National University of Singapore, Singapore; Department of Psychiatry, University of Oxford, Oxford, United Kingdom; Center for Music in the Brain, Department of Clinical Medicine, Aarhus University, Aarhus, Denmark; Life and Health Sciences Research Institute (ICVS), School of Medicine, University of Minho, Braga, Portugal; Centre for Eudaimonia and Human Flourishing, University of Oxford, Oxford, United Kingdom; Department of Information and Communication Technologies, Universitat Pompeu Fabra, Barcelona, Spain; Institució Catalana de la Recerca i Estudis Avancats (ICREA), Barcelona, Spain; Department of Neuropsychology, Max Planck Institute for Human Cognitive and Brain Sciences, Leipzig, Germany; School of Psychological Sciences, Monash University, Melbourne, Australia

**Keywords:** Whole-brain model, Neuroimaging, Exact mean-field model, Hopf bifurcation, Regional heterogeneity

## Abstract

Large variability exists across brain regions in health and disease, considering their cellular and molecular composition, connectivity, and function. Large-scale whole-brain models comprising coupled brain regions provide insights into the underlying dynamics that shape complex patterns of spontaneous brain activity. In particular, biophysically grounded mean-field whole-brain models in the asynchronous regime were used to demonstrate the dynamical consequences of including regional variability. Nevertheless, the role of heterogeneities when brain dynamics are supported by synchronous oscillating state, which is a ubiquitous phenomenon in brain, remains poorly understood. Here, we implemented two models capable of presenting oscillatory behavior with different levels of abstraction: a phenomenological Stuart–Landau model and an exact mean-field model. The fit of these models informed by structural- to functional-weighted MRI signal (T1w/T2w) allowed us to explore the implication of the inclusion of heterogeneities for modeling resting-state fMRI recordings from healthy participants. We found that disease-specific regional functional heterogeneity imposed dynamical consequences within the oscillatory regime in fMRI recordings from neurodegeneration with specific impacts on brain atrophy/structure (Alzheimer’s patients). Overall, we found that models with oscillations perform better when structural and functional regional heterogeneities are considered, showing that phenomenological and biophysical models behave similarly at the brink of the Hopf bifurcation.

## Author Summary

Significant progress has been made in understanding the effects of regional heterogeneity on whole-brain dynamics. With imaging technologies, the number of high-resolution reference maps of brain structure and function has been increased, and whole-brain computational models have provided a suitable avenue to investigate the mechanisms supporting the relations between these maps and whole-brain dynamics. Here, we investigate the role of the heterogeneities when synchronous behavior is present in brain dynamics, which we could represent by models capable of oscillating in the presence of a Hopf bifurcation. We found that models with oscillations more faithfully reproduce empirical properties when structural and functional regional heterogeneities are considered, showing that both phenomenological and biophysical models behave similarly at the brink of the Hopf bifurcation.

## INTRODUCTION

The collective behavior of human brain emerges from the nonlinear dynamics of millions of neurons interacting at billions of synaptic connections (Sporns et al., 2005). Nevertheless, despite this microscopic scale complexity, the brain spontaneously self-organizes into large-scale neuronal networks that give rise to a reduced number of discrete states, where the resting state in particular has been proposed to be a fundamental brain state (Fox, 2005; Gusnard & Raichle, 2001; Raichle & Mintun, 2006). Local and global recordings of the brain have been performed to understand the relation between the neuronal responsiveness and these complex spatiotemporal patterns (Destexhe & Contreras, 2006). In addition, modeling large-scale brain dynamics has been established as a crucial path to unveil mechanisms underlying the emergence of global brain behavior as the interactions of small dynamical units (Deco et al., 2009; Ghosh et al., 2008; Honey et al., 2007).

Whole-brain models based on conceptually simple local dynamical rules coupled according to empirical measurements of anatomical connectivity (Hagmann et al., 2010; Hagmann et al., 2007; Johansen-Berg & Rushworth, 2009) have been successfully implemented to explore the interplay between local dynamics, long-range structural coupling, and the formation of large-scale activity patterns in empirical data (Deco, Cabral, et al., 2017; Deco, Kringelbach, et al., 2017; Ipiña et al., 2020; Jirsa et al., 2002; Piccinini et al., 2021). The local dynamics are described by a set of equations that can be built from knowledge based on biologically realistic mechanisms underlying brain activity, or from neurobiological assumptions concerning the collective dynamics that an ensemble of small units is able to produce (Breakspear, 2017; Cofré et al., 2020; Deco et al., 2008). Several whole-brain models have been proposed based on different local dynamics: from the most abstract such as cellular automata (Haimovici et al., 2013; Tagliazucchi et al., 2016), Ising spin-models (Marinazzo et al., 2014; Ponce-Alvarez et al., 2018), or nonlinear oscillators (Deco, Kringelbach, et al., 2017; Saenger et al., 2017), up to more descriptive models such as neural masses (Breakspear et al., 2003; Honey et al., 2009) or mean-field models (Deco et al., 2014; di Volo et al., 2019; Goldman et al., 2020). However, different sources of empirical data have revealed local variations within each region (Huntenburg et al., 2018), and during the past years whole-brain models based on mean-field local models have been demonstrated to be suitable to unveil how these heterogeneities impact global brain dynamics (Deco et al., 2018; Deco, Kringelbach, et al., 2021; Demirtaş et al., 2019; Kong et al., 2021; P. Wang et al., 2019).

Specifically, the so-called dynamic mean-field whole-brain model (DMFM), as proposed in Deco et al. (2014), was implemented to demonstrate the dynamical consequences of local heterogeneity in reproducing the spatiotemporal structure of empirical data (Demirtaş et al., 2019), its ignition capacity (Deco, Kringelbach, et al., 2021), as well as the role of neuromodulation in altered states of consciousness (Deco et al., 2018). In this model the local activity is represented by a set of differential equations describing the interaction between inhibitory and excitatory pools of neurons (adapted from Wong & Wang, 2006). For each population, three variables are modeled: the synaptic current, the firing rate, and the synaptic gating, where the NMDA and gamma-aminobutyric acid (GABA)-A play a role in the excitatory and inhibitory coupling, respectively. This model is widely used to represent the asynchronous state of global behavior of large ensembles of interconnected neurons, which is guaranteed by the inclusion of a feedback inhibitory control (Deco et al., 2014). Nevertheless, how the heterogeneity shapes whole-brain activity in synchronous states, which is a ubiquitous phenomenon in brain activity, supported by the presence of oscillatory local dynamics is still unclear.

Here, we investigated the dynamical consequences of including regional heterogeneity when brain dynamics are supported by a synchronous state instead of an asynchronous state, as was reported by studies mentioned above. For this purpose, we assessed the impact of regional heterogeneities in the presence of oscillations by implementing whole-brain models capable of representing those synchronous oscillating states. Briefly, we used two models with different levels of abstraction that include a Hopf bifurcation in their local dynamical landscape, which is the simplest representation of both regimes by the same set of equations. The first model is the well-known Stuart–Landau (SL) whole-brain model comprising a set of nonlinear oscillators represented by the normal form of a Hopf bifurcation (Deco, Kringelbach, et al., 2017; Jobst et al., 2017; Saenger et al., 2017; Senden et al., 2017; Wiggins, 2003). The second model is an exact mean-field whole-brain model, which describes the mean activity of a local population of all-to-all coupled quadratic integrate-and-fire (QIF) neurons (Montbrió et al., 2015). Specifically, we fitted both whole-brain models to the large-scale neuroimaging resting-state fMRI data from healthy human participants in the Human Connectome Project (HCP; Van Essen et al., 2013). We demonstrated that both models more faithfully reproduce empirical properties when including a plausible biological source of local heterogeneity, such as regional variation in the ratio of T1- to T2-weighted (T1w/T2w) MRI signal, which is thought to index intracortical myelin content (Glasser & Van Essen, 2011). We tested that this inclusion improves different empirical properties such as the causal ignition (CI), defined as the causal effect that the dynamics of one brain region has on the whole-brain level of synchronization proposed as a quantification of the hierarchical organization of brain function (Deco, Vidaurre, et al., 2021) and static metrics as the functional global brain connectivity (GBC; Deco, Kringelbach, et al., 2021; Demirtaş et al., 2019). Finally, we applied the same framework fitting the SL whole-brain model to neuroimaging resting-state fMRI data from patients with diffuse brain volume heterogeneities (atrophy) triggered by neurodegeneration: Alzheimer’s disease (AD). We found that the inclusion of specifical functional local heterogeneity improves the capacity to reproduce empirical properties, such as functional connectivity, causal ignition, and global brain connectivity.

## METHODS

### Neuroimaging Participants

#### Human Connectome Project data.

The dataset used for this investigation was selected from the March 2017 public data release from the Human Connectome Project (HCP) where we chose a sample of 1,000 participants in resting state. The full informed consent from all participants was obtained by the Washington University–University of Minnesota (WU-Minn HCP) Consortium, and research procedures and ethical guidelines were followed in accordance with Washington University institutional review board approval.

#### Alzheimer’s disease patients’ and controls’ data.

The study comprised 39 patients diagnosed with AD (26 female, 76.6 ± 7 years [mean ± *SD*]). Additionally, a group of 35 healthy controls (38 female, 69.8 ± 7.9 years) was used to compute the functional regional heterogeneity map. An expert neurologist following current criteria diagnosed patients following NINCDS-ADRDA clinical criteria for AD (Dubois et al., 2007; McKhann et al., 2011). Participants were recruited in clinical sites by a multidisciplinary team as part of an ongoing multicentric protocol taking part in the Multi-Partner Consortium to Expand Dementia Research in Latin America (ReDLat) and assessed following harmonized procedures (Ibañez, Fittipaldi, et al., 2021; Ibañez, Pina-Escudero, et al., 2021) as in previous works (Donnelly-Kehoe et al., 2019; Legaz et al., 2022; Melloni et al., 2016; Salamone et al., 2021; Sedeño et al., 2017). Clinical diagnoses were established by neurodegenerative disease experts through an extensive neurological, neuropsychiatric, and neuropsychological examination comprising semi-structured interviews and standardized cognitive assessments. All participants with neurodegenerative conditions were in early/mild stages of the disease. No participant reported a history of other neurological disorders, psychiatric conditions, primary language deficits, or substance abuse. Whole-brain gray matter was compared between AD and controls, showing an extended bilateral temporal with less extended frontoparietal atrophy in AD (Du et al., 2007; Landin-Romero et al., 2017; Pini et al., 2016). All participants provided written informed consent pursuant to the Declaration of Helsinki. The study was approved by the ethics committees of the involved institutions.

#### Brain parcellations.

All neuroimaging data from HCP were processed using two standard cortical parcellations. For a fine-scale parcellation, we used the Glasser parcellation with 360 cortical regions (180 regions in each hemisphere; Glasser et al., 2016). For a coarser scale parcellation, we used the Desikan–Killiany parcellation (Desikan et al., 2006) with a total of 68 cortical regions (34 regions per hemisphere).

### Neuroimaging Acquisition for fMRI

#### Human Connectome Project data.

The HCP website (https://www.humanconnectome.org/) provides the full details of participants and the acquisition protocol of the data for resting state. We used one resting state fMRI acquisition of approximately 15 minutes acquired on the same day of 1003 HCP participants scanned on a 3-T connectome-Skyra scanner (Siemens).

#### Alzheimer’s disease patients’ and controls’ data.

The participants’ data were acquired in three-dimensional volumetric and 10-min-long resting-state MRI sequences. Participants were instructed not to think about anything in particular while remaining still, awake, and with eyes closed. Two independent centers recorded the data, using the parameters described below.

Center 1 (Argentina): Using a 3-T Phillips scanner with a standard head coil, we acquired whole-brain T1-rapid anatomical 3D gradient echo volumes, parallel to the plane connecting the anterior and posterior commissures, with the following parameters: repetition time (TR) = 8,300 ms; echo time (TE) = 3,800 ms; flip angle = 8°; 160 slices, matrix dimension = 224 × 224 × 160; voxel size = 1 mm × 1 mm × 1 mm. Also, functional spin echo volumes, parallel to the anterior-posterior commissures, covering the whole brain, were sequentially and ascendingly acquired with the following parameters: TR = 2,640 ms; TE = 30 ms; flip angle = 90°; 49 slices, matrix dimension = 80 × 80 × 49; voxel size in plane = 3 mm × 3 mm × 3 mm; slice thickness = 3 mm; sequence duration = 10 min; number of volumes = 220. A total of 18 AD patients and 23 controls were scanned in this center.

Center 2 (Chile): Using a 3-T Siemens Skyra scanner with a standard head coil, we acquired whole-brain T1-rapid gradient echo volumes, parallel to the plane connecting the anterior and posterior commissures, with the following parameters: TR = 2,400 ms; TE = 2,000 ms; flip angle = 8°; 192 slices, matrix dimension = 256 × 256 × 192; voxel size = 1 mm × 1 mm × 1 mm. Finally, functional EP2D-BOLD pulse sequences, parallel to the anterior-posterior commissures, covering the whole brain, were acquired sequentially intercalating pair-ascending first with the following fMRI parameters: TR = 2,660 ms; TE = 30 ms; flip angle = 90°; 46 slices, matrix dimension = 76 × 76 × 46; voxel size in plane = 3 mm × 3 mm × 3 mm; slice thickness = 3 mm; sequence duration = 10.5 min; number of volumes = 240. A total of 21 AD patients and 34 controls were scanned in this center.

### Preprocessing and Extraction of Functional Time Series in fMRI Resting Data

#### Human Connectome Project data.

The preprocessing of the HCP resting state is described in detail on the HCP website. In short, the data are preprocessed using the HCP pipeline, which is using standardized methods of FSL (FMRIB Software Library), FreeSurfer, and the Connectome Workbench software (Glasser et al., 2013; Smith et al., 2013). This preprocessing included correction for spatial and gradient distortions and head motion, intensity normalization and bias field removal, registration to the T1-weighted structural image, transformation to the 2-mm Montreal Neurological Institute (MNI) space, and using the FIX artifact removal procedure (Schröder et al., 2015; Smith et al., 2013). The head motion parameters were regressed out and structured artifacts were removed by ICA + FIX processing (independent component analysis followed by FMRIB’s ICA-based X-noiseifier (Griffanti et al., 2014; Salimi-Khorshidi et al., 2014). Preprocessed time series of all grayordinates are in HCP CIFTI grayordinate standard space and available in the surface-based CIFTI file for each participant for resting state.

We used a custom-made MATLAB script using the *ft_read_cifti* function (Fieldtrip toolbox; Oostenveld et al., 2011) to extract the average time series of all the grayordinates in each region of the Schaefer parcellation, which are defined in the HCP CIFTI grayordinate standard space. Furthermore, the BOLD time series were transformed to phase space by filtering the signals with the corresponding TR = 0.72 s in the range between 0.008 Hz and 0.08 Hz (Fox, 2005) and the low-pass cutoff to filter the physiological noise, which tends to dominate the higher frequencies (Cordes et al., 2001; Fox, 2005).

#### Alzheimer’s disease patients’ and controls’ data.

The first five volumes of each fMRI resting-state recording were discarded to ensure a steady state. Data Processing Assistant for Resting-State fMRI (DPARSF V2.3) was used to preprocess the images; DPARSF is an open-access toolbox that generates and implements an automatic pipeline for fMRI analysis within SPM12 and the Resting-State fMRI Data Analysis Toolkit (REST V.1.7). Preprocessing steps included slice-timing correction and realignment to the first scan of the session to correct head movement. Using least squares, we regressed out six motion parameters, as well as cerebrospinal fluid and white matter signals to attenuate the potential effects of residual movement and physiological noise. For this purpose, motion parameters were estimated during the realignment step, and cerebrospinal fluid and white matter masks were obtained from the tissue segmentation of each subject’s T1 scan in native space. None of the participants presented head movements larger than 3 mm and/or rotations higher than 3°, and no differences in head motion among groups were found. As a final step, images were normalized to common MNI space and smoothed using an 8-mm full-width-at-half-maximum isotropic Gaussian kernel. Furthermore, the BOLD time series were transformed to phase space by filtering the signals with the corresponding TR = 2.65 s (rounded for two sites) in the range between 0.008 Hz and 0.08 Hz (Fox, 2005) and the low-pass cut-off to filter the physiological noise, which tends to dominate the higher frequencies (Cordes et al., 2001; Fox, 2005).

### Structural Connectivity Using dMRI

To obtain the structural connectivity we use the Human Connectome Project (HCP) database, which contains diffusion spectrum and T2-weighted imaging data from 32 participants (acquisition parameters are described in detail on the HCP website (Setsompop et al., 2013). Briefly, the data were processed using a generalized q-sampling imaging algorithm implemented in DSI studio (https://dsi-studio.labsolver.org). Segmentation of the T2-weighted anatomical images produced a white matter mask and coregistering the images to the b0 image of the diffusion data using SPM12. In each HCP participant, 200,000 fibers were sampled within the white matter mask. Fibers were transformed into MNI space using Lead-DBS (Horn & Blankenburg, 2016). We used the standardized methods in Lead-DBS to produce the structural connectomes for both the Glasser 360 parcellation (Glasser et al., 2016) and the Desikan–Killiany 68 parcellation (Desikan et al., 2006), where the connectivity has been normalized to a maximum of 0.2. The freely available Lead-DBS software package (https://www.lead-dbs.org/) provides the preprocessing that we implemented and is described in detail in Horn et al. (2017).

### Hopf Whole-Brain Model

**Figure 1.**
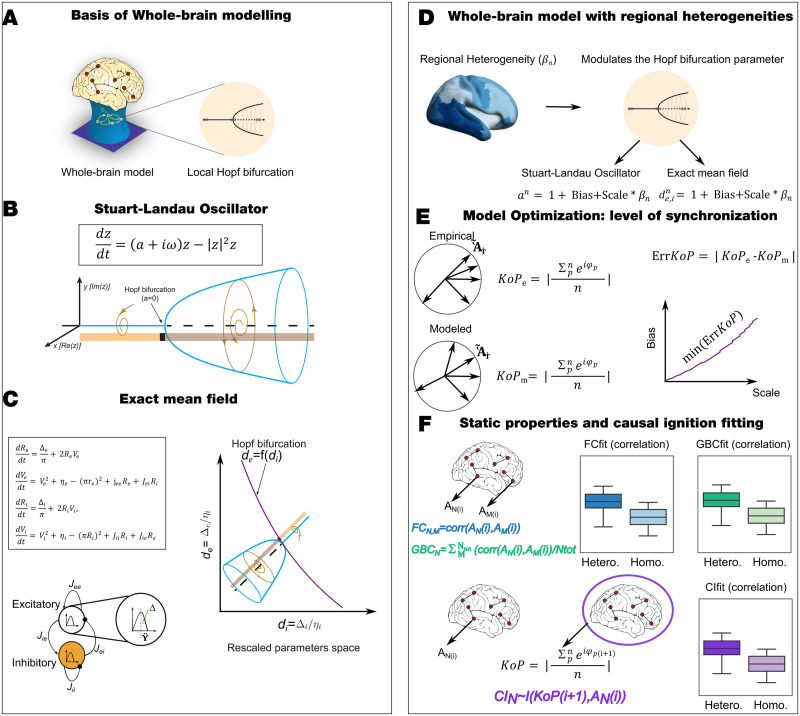
Overview of framework. (A) Whole-brain models integrate anatomy and local dynamics that in our work present a Hopf bifurcation, that is, the capability to change the dynamical behavior from a fixed point governed by noise toward sustained oscillations. These whole-brain models are able to reproduce static and dynamic properties computed from empirical fMRI data. (B) The Stuart–Landau equation is the simplest representation of a Hopf bifurcation and is suited for describing the transitions between noise and oscillation by varying only one bifurcation parameter, *a*. (C) The exact mean-field model is derived from neuronal quadratic integrate-and-fire equations in the thermodynamic limit (i.e., large number of neurons). The resulting firing rate equations (FRE) describe the dynamics of the mean firing rate (*R*) and the mean membrane potential (*V*) of a heterogeneous population of all-to-all coupled neurons. The heterogeneity within each population of neurons lies in the input current that each neuron received and is described by a Lorentzian distribution of half-width Δ and center *η*. The generalization of a pool of interacting inhibitory and excitatory populations includes an equation for the mean firing rate and mean membrane potential for each population and a coupling term modulated by the mean synaptic strength factor, *J*. We reduced the number of parameters of the system by the nondimensionalization of FRE equations rescaling and redefining the variables and parameters. The dynamical landscape of an interacting population of inhibitory and excitatory neurons presents a Hopf bifurcation in two-dimensional phase diagram determined by the rescaled half-width of the Lorentzian distribution *d*_*e*−_ = Δ_*e*_/*η*_*i*_ − *d*_*i*−_ = Δ_*i*_/*η*_*i*_, which are named bifurcation parameters, with other model parameters fixed (*η* = $\frac{40}{30}$; *j*_*ee*_ = $\frac{50}{\sqrt{40}}$; *j*_*ie*_ = $\frac{20}{\sqrt{40}}$; *j*_*ie*_ = *j*_*ii*_ = −$\frac{20}{\sqrt{40}}$). (D) The regional heterogeneity modulates the bifurcation parameter, *a*, in the Hopf whole-brain model, and the parameters *d*_*e*_ and *d*_*i*_ in the exact mean-field whole-brain model. The modulation consists of an additive term, named the bias, and a term that is a factor multiplying the regional heterogeneity, called scale. (E) We fit the homogeneous model to the empirical level of synchronization measures as the mean of the modulus of the Kuramoto order parameter (KoP) computed by the absolute difference between the empirical and the modeled values (ErrKoP). The bias and scale are explored and a curve of the minimum level of ErrKoP is determined. (F) We compute the model fit capacity to other static and dynamic properties along the curve of iso-level of minimum ErrKoP. We find that for all the measures, the heterogeneous model outperforms the homogeneous model.

Whole-brain models have been largely used to describe the most important features of the actual brain activity. These models provide a balance between complexity and realism by representing the macroscopic brain dynamics as an emergent behavior of millions of small interacting units. One of the macroscopic dynamical features is that the collective behavior dynamics can range from a fully synchronous to a stable asynchronous state governed by random fluctuations. The simplest dynamical system capable of presenting both behaviors is the one described by a Stuart–Landau nonlinear oscillator, which is mathematically described by the normal form of a supercritical Hopf bifurcation:

$$\frac{dz}{dt}=\left(a+i\omega \right)z-z{\left|z\right|}^{2},$$ (1)

where *z* is a complex-valued variable (*z* = *x* + *iy*), and *ω* is the intrinsic frequency of the oscillator. The bifurcation parameter *a* qualitatively changes the nature of the solutions of the system. Specifically, at *a* = 0, a so-called Hopf bifurcation occurs, so that for *a* > 0 (supercritical regime) the system has a stable limit cycle (and hence displays self-sustained oscillations) and an unstable fixed point. In contrast, for *a* < 0 (subcritical regime) only a stable fixed point (focus) exists (Figure 1B).

The coordinated dynamics of the resting-state activity are modeled by introducing coupling between these oscillators. Several works have demonstrated that whole-brain models based on Stuart–Landau oscillators ruling the local dynamical behavior have the capability to describe different empirical observables from brain recordings when the oscillators are coupled with the structural connectivity (Deco, Kringelbach, et al., 2017; Hansen et al., 2015; Jobst et al., 2017).

The dynamical of region *i* in the coupled whole-brain system is described in Cartesian coordinates:

$$\frac{dRe\left(z_{i}\right)}{dt}=\frac{dx_{i}}{dt}=a_{i}x_{i}-\left[x_{i}^{2}+y_{i}^{2}\right]x_{i}-\omega_{i}y_{i}+G\sum_{j=1}^{N}C_{ij}\left(x_{j}\left(t\right)-x_{i}\right)+\nu_{i}\eta_{i}\left(t\right),$$

$$\frac{dIm\left(z_{i}\right)}{dt}=\frac{dy_{i}}{dt}=a_{i}y_{i}-\left[x_{i}^{2}+y_{i}^{2}\right]y_{i}+\omega_{i}x_{i}+G\sum_{j=1}^{N}C_{ij}\left(y_{j}\left(t\right)-y_{i}\right)+\nu_{i}\eta_{i}\left(t\right),$$ (2)

where *η*_*i*_(*t*) is an additive Gaussian noise with standard deviation *ν*, *G* is a factor that scales the strength of the coupling equally for all the nodes, and *x*_*j*_ is the dynamical variable that simulates the fMRI signal of region *j*.

To fit empirical data to the homogeneous model, we exhaustively explore the coupling strength, *G*, with the bifurcation parameter, *a*_*n*_, fixed equally for all the brain regions (*a* = −0.02). We then fit empirical data to the heterogenous model; we modulate the local bifurcation parameter by the inclusion of the regional heterogeneity as follows:

$$a^{n}=1+\delta_{1}+\delta_{2}\times \beta_{n},$$ (3)

where *δ*_1_ and *δ*_2_ stand for the bias and scale, respectively, that modulate the regional heterogeneity *β*_*n*_. The regional heterogeneity during the first part of this work is provided by the T1w/T2w ratio and in the second part by the node-level functional connectivity.

### Exact Mean-Field Whole-Brain Model

We implement a whole-brain model based on local mean-field equations. These mean-field equations are exactly derived from a large population of all-to-all coupled quadratic integrate-and-fire (QIF) neurons (Devalle et al., 2017; Montbrió et al., 2015). The microscopic state of the population of QIF neurons is determined by the membrane potential of each neuron *i*, *V*_*i*_, which evolves according to

$$\tau_{m}\frac{dV_{i}}{dt}=V_{i}^{2}+I_{i},\;\left(\text{where}\;i=1,\ldots ,N\right),$$ (4)

where *τ*_*m*_ is the membrane time constant. The resetting rule of the QIF neurons is as follows: Each time a neuron’s membrane potential reaches the peak value *V*_*th*_, the neuron emits a spike and its voltage is reset to the value *V*_*r*_. The membrane time constant is *τ*_*m*_ = 10 ms, and *I*_*i*_ represents the input current to the neuron *i*, which is the following:

$$I_{i}=\eta_{i}+J\tau_{m}\;R\left(t\right).$$ (5)

Here *η*_*i*_ is a heterogeneous current (taken from a Lorentzian distribution; see Equation 6), and the last term represents recurrent coupling of strength *J*, which is mediated by the mean firing rate of the population, *R*(*t*).

To derive the exact mean-field model corresponding to a population of QIF neurons, one needs to adopt the thermodynamic limit (*N* tends to infinity) and consider *V*_*th*_ = −*V*_*r*_ = ∞. Finally, considering a Lorentzian distribution of input currents centered in *η*, and half-width Δ (see Figure 1C),

$$g\left(\eta \right)=\frac{1}{\pi }\frac{\Delta }{{\left(\eta -\bar{\eta }\right)}^{2}+\Delta^{2}},$$ (6)

one finds the so-called firing rate equations (FRE) corresponding to the QIF network (Devalle et al., 2017; Montbrió et al., 2015):

$$\tau_{m}\frac{dR}{dt}=\frac{\Delta }{\pi \tau_{m}}+2RV,$$

$$\tau_{m}\frac{dV}{dt}=V^{2}+\bar{\eta }-{\left(\pi \tau_{m}R\right)}^{2}+J\tau_{m}R,$$ (7)

where *R* is the mean firing rate of the population of QIF neurons, and *V* its mean membrane potential.

We considered two populations of interacting excitatory (*e*) and inhibitory (*i*) neurons, and we build a whole-brain model by including large-scale interconnections between these local populations of neurons according to the structural connectivity matrix. The inter-area connections, which are established as long-range excitatory synaptic connections, and the intra-area connection between excitatory-excitatory pools are weighted by the strength synaptic. At the same time the coupling terms are scaling by a global scaling factor *G*, which denotes the density of fibers between those regions. The global scaling factor is a free control parameter. The dynamics of brain region *n* modeled as these two interacting populations coupled with the rest of the brain are described by the following:

$$\tau_{m}\frac{dR_{e}^{n}}{dt}=\frac{\Delta_{e}}{\pi \tau_{m}}+2R_{e}^{n}V_{e}^{n},$$

$$\tau_{m}\frac{dV_{e}^{n}}{dt}={\left(V_{e}^{n}\right)}^{2}+\eta_{e}-{\left(\pi \tau_{m}R_{e}^{n}\right)}^{2}+J_{ee}\tau_{m}R_{e}^{n}+J_{ei}\tau_{m}R_{i}^{n}+GJ_{ee}\tau_{m}\sum_{p=1}^{N}C_{np}R_{e}^{p},$$

$$\tau_{m}\frac{dR_{i}^{n}}{dt}=\frac{\Delta_{i}}{\pi \tau_{m}}+2R_{i}^{n}V_{i}^{n},$$

$$\tau_{m}\frac{dV_{i}^{n}}{dt}={\left(V_{i}^{n}\right)}^{2}+\eta_{i}-{\left(\pi \tau_{m}R_{i}^{n}\right)}^{2}+J_{ii}\tau_{m}R_{i}^{n}+J_{ie}\tau_{m}R_{i}^{n},$$ (8)

where *J*_*xx*_ stands for the mean synaptic strength for the cases of excitatory to excitatory (*xx* = *ee*), inhibitory to excitatory (*xx* = *ei*), inhibitory to inhibitory (*xx* = *ii*), and excitatory to inhibitory (*xx* = *ie*). The coupling between the areas *n* and the brain region *p* occurs only at the E-to-E level and is scaled by the structural connectivity *C*_*np*_ (see above sections in Methods).

### Nondimensionalized Exact Mean-Field Whole-Brain Model

The equations of the full coupled exact mean-field whole-brain model have five parameters for each population and the coupling strength parameter. Following Devalle et al. (2017), it is possible to nondimensionalize these equations so that the system can be written solely in terms of six parameters. Generally, we adopt the following notation: We use capital letters to refer to the original variables and parameters of the exact mean-field model, and lowercase letters for nondimensional quantities.

We define the rescaled variables as

$$r_{e,i}=\frac{\tau_{m}R_{e,i}}{\sqrt{\eta_{i}}},\;v_{e,i}=\frac{V_{e,i}}{\sqrt{\eta_{i}}},\;\tilde{t}=\frac{\sqrt{\eta_{i}}}{\tau_{m}}t,$$ (9)

and the nondimensionalized parameters as

$$j_{xx}=\frac{J_{xx}}{\sqrt{\eta_{i}}},\;d_{e,i}=\frac{\Delta_{e,i}}{\eta_{i}},\;\eta =\frac{\eta_{e}}{\eta_{i}}.$$ (10)

Then the equations of the nondimensional exact mean-field whole-brain model can be written as follows:

$$r_{e}^{\prime }=\frac{d_{e}}{\pi }+2r_{e}v_{e},$$

$$v_{e}^{\prime }=v_{e}^{2}+\eta -{\left(\pi r_{e}\right)}^{2}+j_{ee}r_{e}+j_{ei}r_{i}+Gj_{ee}\sum_{p=1}^{N}C_{np}r_{e}^{p},$$

$$r_{i}^{\prime }=\frac{d_{i}}{\pi }+2r_{i}v_{i},$$

$$v_{i}^{\prime }=v_{i}^{2}+1-{\left(\pi r_{i}\right)}^{2}+j_{ii}r_{i}+j_{ie}r_{e}.$$ (11)

To compare with fMRI empirical observables, the simulated firing activity of each excitatory pool of neurons was transformed into BOLD-like signals with the same TR as the empirical data using the Balloon–Windkessel model (Deco et al., 2014; Stephan et al., 2007). We fit the homogeneous model by exploring the bidimensional parameter space defined by the coupled strength, *G*, and the half-wide of the inhibitory population, *d*_*i*_, with other model parameters constant (*η* = $\frac{40}{30}$; *j*_*ee*_ = $\frac{50}{\sqrt{40}}$; *j*_*ie*_ = $\frac{20}{\sqrt{40}}$; *j*_*ie*_ = *j*_*ii*_ = −$\frac{20}{\sqrt{40}}$). We then include the regional heterogeneity as bias (*δ*_1_) and scale (*δ*_2_) terms modulating the T1w/T2w ratio which modifies equally the half-width of the intra-region heterogeneity distribution of *d*_*i*_, *d*_*e*_:

$$d_{e,i}^{n}=1+\delta_{1}+\delta_{2}\times \beta_{n}.$$ (12)

### Functional Connectivity Computation and Fitting

The functional connectivity (FC) is defined as the *N* × *N* matrix of BOLD signal time correlations between brain areas computed over the entire recording period, where *N* is the number of brain regions in the corresponding brain parcellations. We compute the empirical FC for each human participant and for each simulated trial (the total number of trials matched the number of participants). The group-averaged empirical and modeled FC matrices were compared by computing the Pearson correlation between their upper triangular elements (given that the FC matrices are symmetric):

$$\text{FCfit}=\text{corr}\left({FC}_{emp},{FC}_{\mathrm{mod}}\right).$$ (13)

### Global Brain Connectivity Computation and Fitting

We compute the global brain connectivity (GBC) as was defined in Demirtaş et al. (2019); it stands for another static spatial metric characterizing the average FC strength for each area. It has also been called static-node level in previous work (Deco, Kringelbach, et al., 2021). Mathematically, the GBC for each brain region *i* is computed as follows:

$${GBC}_{i}=\frac{\sum_{j=1}^{N}{FC}_{ij}}{N},$$ (14)

where *FC*_*ij*_ are the elements *I* and *j* of the FC matrix and *N* represents the numbers of considered brain regions. We compute the empirical GBC vectors for each human participant and for each simulated trial (the total number of trials matched the number of participants). The GBCfit is quantified by computing the Pearson correlation between the group-averaged empirical and modeled GBC vectors.

$$\text{GBCfit}=\text{corr}\left({GBC}_{emp,}{GBC}_{\mathrm{mod}}\right).$$ (15)

### Kuramoto Order Parameter Computation and Fitting

The level of global synchronization of a system of *N* oscillators is usually measured by the Kuramoto order parameter (KoP). If the system is completely independent, the *n* phases are uniformly distributed, and thus KoP is nearly zero, while KoP = 1 if all phases are equal (full synchronization). For calculating KoP of the empirical and simulated BOLD signals, we first band-pass filtered within the narrowband 0.008–0.08 Hz and computed the instantaneous phase *φ*_*k*_(*t*) of each narrowband signal of the region *k* using the Hilbert transform. The Hilbert transform yields the associated analytical signals. The analytic signal represents a narrowband signal, *s*(*t*), in the time domain as a rotating vector with an instantaneous phase, *φ*_*k*_(*t*), and an instantaneous amplitude, *A*(*t*). We compute the KoP for the whole-brain activity in a parcellation including *N* brain regions as follows:

$$KoP=\left|\frac{\sum_{p}^{N}e^{i\varphi_{p}\left(t\right)}}{N}\right|.$$ (16)

We compute the empirical KoP for each human participant and for each simulated trial (the total number of trials matched the number of participants) and then average it across subjects. We measure the level of fitting by computing the absolute difference between the empirical and the modeled KoP group average:

$$\text{ErrKoP}=abs\left({KoP}_{emp}-{KoP}_{\mathrm{mod}}\right).$$ (17)

### Causal Ignition Computation and Fitting

We define the causal ignition based on the previous work in which Deco and colleagues established a novel framework named normalized directed transfer entropy (NDTE) to characterize how different brain regions communicate with each other in terms of information-theoretical statistical criterion (Deco, Vidaurre, et al., 2021). This framework uses only the second-order statistics of the involved entropies, which means that instead of estimating the probabilities, the method estimates the covariance, which massively facilitates computation. Then, four computations are performed: normalization, multiple time points in the past, circular surrogates, and aggregation of *p* values to improve the reliability and robustness of the NDTE framework.

We implement the NDTE framework to compute instead the causality relationship between brain area activity, the causality between the amplitude of the oscillation of one brain region *n* (*A*_*n*_), and the level of brain global synchronization (KoP). To do so, we measure the extra knowledge that the dynamical activity of the past of *A*_*n*_ contributes to the prediction of the future of KoP, by the following mutual information:

$$I_{n}\left({KoP}_{i+1};{A_{n}}^{i}|{KoP}^{i}\right)=H\left({KoP}_{i+1}|{KoP}^{i}\right)-H_{n}\left({KoP}_{i+1}|{A_{n}}^{i},{KoP}^{i}\right),$$ (18)

where *KoP*_*i*+1_ is the level of brain synchronization measured by the Kuramoto order parameter at time point *i* + 1, and *A*_*n*_^*i*^ indicates the amplitude of the brain region *n* of the past of *A*_*n*_ (computed by the Hilbert transform of the filtered BOLD signal, as was explained above, sampled in repetition time, TR) in a time window of length *T* up to and including the time point *i* (we considered *T* = 10). It is remarkable that *I*_*n*_(*KoP*_*i*+1_; *A*_*n*_^*i*^|*KoP*_*i*_) expresses a strong form of Granger causality (Granger, 1980), by comparing the uncertainty in *KoP*_*i*+1_ when using knowledge of only its own past *KoP*_*i*_ or the past of both; that is, *A*_*n*_^*i*^, *KoP*_*i*_. This information-theoretical concept of causality was introduced in neuroscience by Schreiber (2000) and is usually called the transfer entropy (Brovelli et al., 2015; Vicente et al., 2011). Brovelli et al. (2015) proposed a weaker form of causality, allowing the calculation of the involved entropies by just considering a Gaussian approximation. Under this approximation, the entropies can be computed as follows:

$$H\left({KoP}^{i}\right)=\frac{T}{2}\log\left(2\pi e\right)+\frac{1}{2}\log\left(\det\left(\sum \left({KoP}^{i}\right)\right)\right),$$

$$H\left({KoP}^{i+1},{KoP}^{i}\right)=\frac{T+1}{2}\log\left(2\pi e\right)+\frac{1}{2}\log\left(\det\left(\sum \left({KoP}^{i+1},{KoP}^{i}\right)\right)\right),$$

$$H_{n}\left(A_{n}^{i},{KoP}^{i}\right)=T\log\left(2\pi e\right)+\frac{1}{2}\log\left(\det\left(\sum \left({A_{n}}^{i},{KoP}^{i}\right)\right)\right),$$

$$H\left({KoP}^{i+1},{KoP}^{i},A_{n}^{i}\right)=\frac{2T+1}{2}\log\left(2\pi e\right)+\frac{1}{2}\log\left(\det\left(\sum \left({KoP}^{i+1},{KoP}^{i},A_{n}^{i}\right)\right)\right).$$ (19)

In this way we are able to compute the direct causality between the activity of one region and the global level of synchronization. To be able to compare mutual information between each node the metrics are normalized as follows:

$${CI}_{KoP,A_{n}}=I_{n}\left({KoP}_{i+1};{A_{n}}^{i}|{KoP}^{i}\right)/I_{n}\left({KoP}_{i+1};{A_{n}}^{i},{KoP}_{i}\right),$$ (20)

where the denominator stands for the mutual information that the past of the amplitude and global synchronization have about the future of the global of level of synchronization. We obtain the CI as a vector of size *N* equal to the brain regions in the considered parcellation.

Finally, following the NDTE framework, we perform statistical significance analysis of the CI by implementing a surrogate framework. Briefly, we use the circular shift methodology for analyzing the *p* values of the hypothesis testing, aiming to detect significant values in CI for each region for each single participant (for further details in the surrogate creation and rational, see Deco, Vidaurre, et al., 2021). After computing these individual *p* values, we aggregate the *p* values for each brain area across the whole group of participants. The combination of different *p* values across subjects is addressed by implementing the Fisher’s method (Fisher, 1992). Here, we use a more sensitive methodology, namely, the Stouffer’s method (Stouffer et al., 1949), which sums the inverse normal transformed *p* values. After the aggregation of the pairs of *p* values across participants, we correct for multiple comparisons by using the traditional false discovery rate method of Benjamini and Hochberg (Hochberg & Benjamini, 1990). The result of the significance test across participants determines a binary vector *M* (with the dimension being the number of brain regions in a given parcellation) that indicates with ones or zeros whether the corresponding brain region is significant.

We compute the empirical CI for each human participant and for each simulated trial (the total number of trials matched the number of participants), masked by the empirical vector *M*, and then average it across subjects. We measure the level of fitting by computing the correlation between the empirical and the modeled masked CI group average:

$$\text{CIfit}=\text{corr}\left({CI}_{emp},{CI}_{\mathrm{mod}}\right).$$ (21)

The framework described above requires large-scale high-resolution sampling rate data, and thus the computation of CI directly to the Alzheimer’s dataset was not possible. For that reason we define a simple version, as a proxy of the CI computed as the correlation between the amplitude of the region, *n*, in time *t*, *A*_*n*_(*t*), and the global level of synchronization in the next time step, *KoP*(*t* + 1).

## RESULTS

### Overview

Our overall framework is schematically displayed in Figure 1 and is explained in detail in the Methods section. Briefly, we used whole-brain models that combine different sources of empirical information in order to replicate static and dynamic empirical properties observed in fMRI recordings. In this work, we focused on models where local dynamics are described by a Hopf bifurcation, that is, the capability to change the dynamic behavior from a fixed point governed by noise toward sustained oscillations. Specifically, we investigated two models with different levels of abstraction near the Hopf bifurcation point: a widely investigated phenomenological model built by coupled nonlinear Stuart–Landau oscillators (Deco & Jirsa, 2012; Deco, Kringelbach, et al., 2017; Deco et al., 2014; Freyer et al., 2012; Ghosh et al., 2008; Honey et al., 2007; Figure 1B); and an exact mean-field model, consisting of a system of two coupled ordinary differential equations (Montbrió et al., 2015; Figure 1C). This system of equations—often referred to as firing rate equations (FRE)—exactly describes the dynamics of the mean firing rate (*R*) and the mean membrane potential (*V*) of a large, heterogeneous population of all-to-all coupled QIF neurons. The heterogeneity within each population is represented by the half-width Δ and center *η* of the Lorentzian distribution standing for the neurons’ input currents, which are necessary for the exact derivation of the mean-field model. Consequently, the FRE corresponding to one population depends on only these parameters, and thus the mean firing rate and mean membrane potential dynamics are determined by the level of heterogeneity within the population (Devalle et al., 2017; see the Methods section). We numerically explored the dynamical landscape of an interacting pair of populations, one inhibitory and one excitatory, following the FRE derived by Montbrió et al. (2015), after performing a nondimensionalization of the FRE (Devalle et al., 2017; see the Methods section). For this exploration, we initially considered model parameters based on previous studies (Devalle et al., 2017; Montbrió et al., 2015), and we then tuned these values to obtain the two-dimensional parameter space determined by *d*_e_ − *d*_i_ (the rescaled half-width of the excitatory and inhibitory population, whereas the other parameters were fixed: *η* = $\frac{40}{30}$; *j*_*ee*_ = $\frac{50}{\sqrt{40}}$; *j*_*ie*_ = $\frac{20}{\sqrt{40}}$; *j*_*ie*_ = *j*_*ii*_ = −$\frac{20}{\sqrt{40}}$) a Hopf bifurcation (representing excitation-inhibition-based oscillations, purple line in the phase diagram in Figure 1C). We included regional heterogeneity to modulate the bifurcation parameter, *a*, in the Hopf whole-brain model and the parameters *d*_e_ and *d*_i_ in the exact mean-field whole-brain model. The modulation was the same for both models, consisting of an additive term (referred to as the “bias”), and a term that is a constant multiplied by the regional heterogeneity (referred to as “scale”; Figure 1D).

First, we fitted the homogeneous model, that is, with zero bias and zero scale, to the empirical level of synchronization, quantified as the mean of the modulus of the Kuramoto order parameter (KoP). The quality of fit was defined as the absolute difference between the empirical and the modeled values (ErrKoP). Second, on top of the homogeneous model we exhaustively explored the values of the bias and scale, and determined a curve of minimum level of ErrKoP in the bias-scale parameter space (Figure 1E). Finally, we computed the model fit capacity to other static and dynamical properties along the iso-level curve of minimum ErrKoP. We evaluated the performance of the model with respect to some of the major outcome measures evaluated thus far in the literature; namely, the static FC and the GBC, but also with respect to a measure that quantifies the causal effect that each brain region has on the whole-brain of synchronization. To do so, we considered the dynamics of one brain region at a certain time *i* and then computed the causal effect that it has on the whole-brain level of synchronization in time *i* + 1, in terms of Granger causality (Figure 1F; see the Methods section for a more detailed explanation).

### Dynamical Consequences of Regional Heterogeneity in the Stuart–Landau Whole-Brain Model

**Figure 2.**
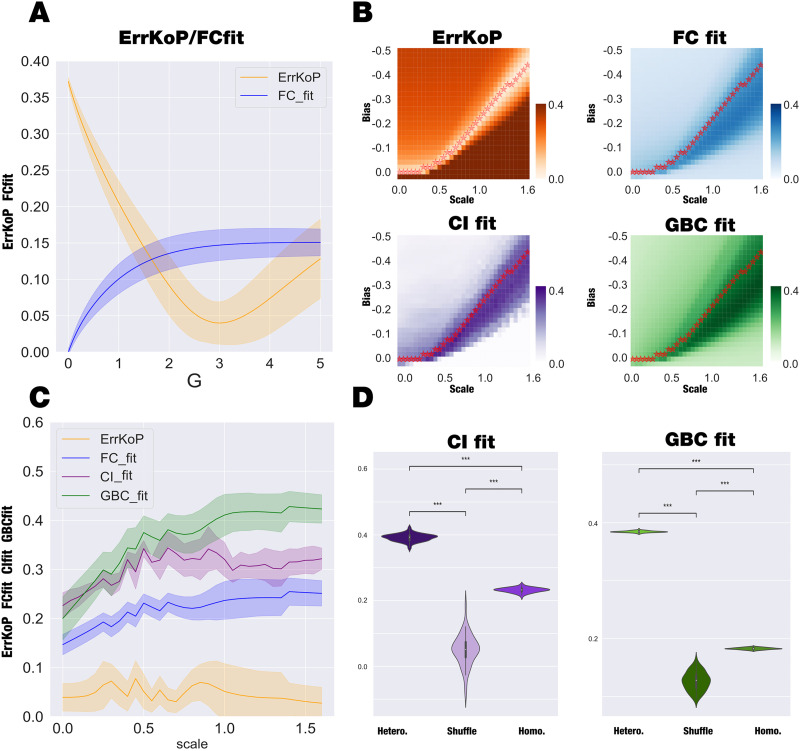
Hopf whole-brain model and the impact of regional heterogeneity T1w/T2w ratio. (A) The homogeneous model (*a* = −0.02 for all nodes) was fitted to the empirical data by computing the correlation between model and empirical functional connectivity (FCfit, blue) and absolute error of the model and empirical Kuramoto order parameter (ErrKoP, orange) as a function of the global coupling parameter, *G*. Only the fit to ErrKoP shows a clear optimum at *G* = 3.23, while the FC fitting level asymptotically reaches a maximum. (B) Using the optimal working point found for the homogeneous model, we introduced regional heterogeneity and assessed its impact by exploring the two-dimensional space determined by the bias and the scaling that directly modify the regional bifurcation parameters. We computed four different fitting measures: the ErrKoP and the FC, as in the homogeneous case, but also the global brain connectivity fit (GBC) and the error in Granger causality (CIfit), both computed as the Pearson correlation between the empirical and simulated measures. We defined an iso-level curve of ErrKoP, represented by red stars in the four matrices, computed as the value of bias that reaches the minimum value of ErrKoP for each value of scaling. (C) Across this iso-ErrKoP curve we computed the value of the four observables computed in panel B. Considering that the pair (0, 0) stands for the homogeneous case, we observed that CIfit, GBCfit, and FCfit increase with the inclusion of the heterogeneity (the ErrKoP remains constant by definition in this plot). (D) We selected the scale value where the maximal CIfit is reached and computed 50 times the CIfit and the GBCfit with the regional heterogeneity, with the homogeneous model, and with a spatial null model (generated by shuffling the regions of heterogeneity, preserving the spatial autocorrelation). The boxplots show the comparison of the three models in the two measures, presenting statistical significance for all the cases (*** *P* < 0.001, Wilcoxon rank sum test with Bonferroni correction).

We assessed the homogeneous SL whole-brain model performance in reproducing empirical properties of resting-state data in a parcellation comprising 360 cortical areas (Glasser et al., 2016). We explored the global coupling parameter *G* with all regional bifurcation parameter *a*_*i*_ = −0.02 (i.e., heterogeneity is not considered). Figure 2A shows how well the model fits, as a function of *G*, by computing the correlation between model and empirical functional connectivity (FCfit) and absolute error of the model and empirical Kuramoto order parameter (ErrKoP) (see Methods for further details). We selected as the homogeneous model working point the minimum of ErrKoP, which shows a clear optimum at *G* = 3.23, while the FC fitting level asymptotically reaches a maximum.

We then studied how regional heterogeneity given by the T1w/T2w affects the fitting of the Kuramoto order parameter (ErrKoP) and the functional connectivity (FCfit). We also considered the fitting of causal ignition (CI) and global brain connectivity (GBC), both computed as the Pearson correlation between the empirical and simulated measures, referred to as CIfit and GBCfit. The CI metric is a measure of local causality reflecting how the activity of one brain region affects the whole-brain dynamics, while the GBC is a local metric that indicates how each brain region is functionally connected to the rest of the brain. We introduced this heterogeneity by modulating the regional bifurcation parameter *a*_*i*_, at the optimal working point of the homogeneous model (*G* = 3.23), by a multiplicative factor, named scale, and additive term, named bias. Figure 2B shows the evolution of the fitting of the four measures as a function of the scale and bias. We identified an iso-level curve of ErrKoP, represented by red stars in the four matrices, computed as the value of bias that reaches the minimum value of ErrKoP for each value of scaling. Figure 2C presents the level of fitting of these four measures along the iso-level curve of ErrKoP starting from zero scale and bias, that is, the homogeneous case. We defined as the heterogeneous model’s optimum working point the scale and bias values where the maximal CIfit is reached within the iso-level ErrKoP curve (i.e., the maximum of the CIfit, purple, curve in Figure 2C). The distributions of fit statistics of CIfit and GBCfit across 50 runs of the homogenous model, heterogeneous model, and null hypothesis generated by spatially shuffling the regional heterogeneity are displayed in Figure 2D.

The spatial shuffling was carefully generated to preserve the spatial autocorrelation of the heterogeneous map, following the work of Burt et al. (2020). In the Supporting Information, Figure S1, we displayed the map and its corresponding surrogates. The heterogeneous model presents a significant improvement in the fitting capacity compared with the homogenous model and shuffled control (*P* < 0.001, Wilcoxon rank sum test with Bonferroni correction in all the comparisons). In summary, we can conclude that the inclusion of structural regional heterogeneities improves the model fitting beyond the inclusion of more model parameters (the shuffled control presents the same number of parameters as the heterogeneous case without improvements in the model-fitting capacity).

### Dynamical Consequences of Regional Heterogeneity in Exact Mean-Field Whole-Brain Model

**Figure 3.**
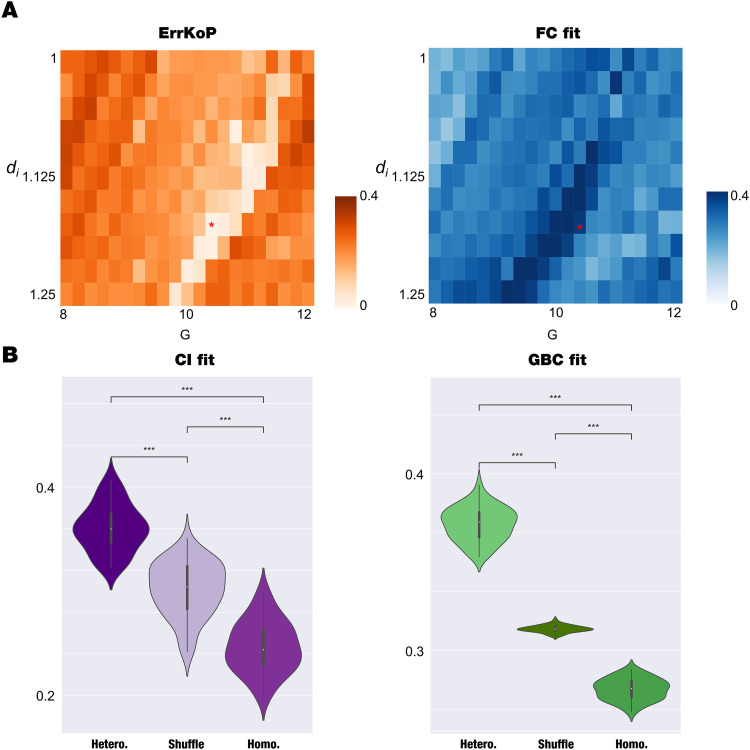
Exact mean-field whole-brain model and the impact of regional heterogeneity T1w/T2w ratio. (A) The homogeneous model (*η* = $\frac{40}{30}$; *j*_*ee*_ = $\frac{50}{\sqrt{40}}$; *j*_*ie*_ = $\frac{20}{\sqrt{40}}$; *j*_*ie*_ = *j*_*ii*_ = −$\frac{20}{\sqrt{40}}$ equally for all nodes) was fitted to the empirical data by computing the correlation between model and empirical functional connectivity (FCfit, blue) and absolute error of the model and empirical Kuramoto order parameter (ErrKoP, orange) as a function of the global coupling parameter, *G*, and the rescaled dispersion of the local level of heterogeneity of inhibitory population, *d*_*i*_. We selected the minimum value of ErrKoP as the optimal working point of the homogenous model found at *G* = 1.04 and *d*_*i*_ = 1.175, indicated with red stars in both matrices. (B) We explored the impact of local T1w/T2w ratio heterogeneity in this model through the inclusion of two parameters, the scaling and bias, which equally modify *d*_*i*_ and *d*_*e*_, and computed the GBCfit and CIfit for each pair. For each scale we found the bias that presents the minimum value of ErrKoP, and we selected the best heterogeneous fit as the one that maximizes the fitting of CIfit at scale = 2.1 and bias = −0.7. We computed 50 times the CIfit and the GBCfit with the regional heterogeneity at that optimal point, with the homogeneous model (scale = 0; bias = 0) and a spatial null model (generated by shuffling the regions of heterogeneity, preserving the spatial autocorrelation). The boxplots show the comparison of the three models in the two measures, presenting statistical significance for all the cases (*** *P* < 0.001, Wilcoxon rank sum test with Bonferroni correction).

We evaluated the homogeneous exact mean-field model performance in reproducing empirical properties of the resting state. For a coarser scale parcellation suitable for mean-field whole-brain computational modeling, we used a Desikan–Killiany parcellation comprising 68 cortical areas (Desikan et al., 2006). We explored two parameters: the global coupling parameter, *G*, and the rescaled dispersion of the local level of heterogeneity of inhibitory population, *d*_*i*_, with the other model parameters equal to all regions (*η* = $\frac{40}{30}$; *d*_*e*_ = 1; *j*_*ee*_ = $\frac{50}{\sqrt{40}}$; *j*_*ie*_ = $\frac{20}{\sqrt{40}}$; *j*_*ie*_ = *j*_*ii*_ = −$\frac{20}{\sqrt{40}}$) to be near the Hopf bifurcation (see Figure 1 and the Methods section). It is remarkable that we have initially explored the parameter corresponding to the inhibitory populations following previous work that reveals the importance of the inhibitory network in facilitating stable macroscopic emergent dynamics in the brain (Hellyer et al., 2016), in providing a mechanism for brain oscillatory dynamics (Devalle et al., 2017), and in compensating the excess of long-range excitatory connections (Deco et al., 2014). To compare with fMRI empirical observables, the simulated firing activity of each excitatory pool of neurons was transformed into BOLD-like signals using the Balloon–Windkessel model (Deco et al., 2014; Stephan et al., 2007). We fitted the model to empirical data by computing the correlation between the model and empirical functional connectivity (FCfit, blue) and absolute error of the model and empirical Kuramoto order parameter (ErrKoP, orange). To do so, we exhaustively explored the parameter space defined by the global coupling parameter, *G*, and the dispersion of the local heterogeneity level of inhibitory population, *d*_*i*_ (Figure 3A). We selected the minimum value of ErrKoP as the optimal working point of the homogenous model found at *G* = 1.04 and *d*_*i*_ = 1.175, indicated with red stars in both matrices in Figure 3A. We replicated these results by exploring the level of heterogeneity in the excitatory population (*d*_*e*_) and fixed the value of the inhibitory population (*d*_*i*_ = 1). The exploration shows a similar structure to fit the synchronicity and functional connectivity (compared with Figure 3A), presenting a similar optimum working point with similar fitting levels (see the Supporting Information, Figure S2). This result is aligned with the similar role these parameters play in the dynamical system, as seen in the diagram with the Hopf bifurcation displayed in Figure 1.

We then explored the impact of local T1w/T2w ratio heterogeneity in this model through the inclusion of two parameters, the scaling and bias, which equally modify *d*_*i*_ and *d*_*e*_, and computed the GBCfit and CIfit for each bias-scale pair. Following the procedure that we performed with the phenomenological model, we found for each scale the bias that yields the minimum ErrKoP value, and we selected the best heterogeneous fit as the one that maximizes the fitting of CIfit (scale = 2.1 and bias = −0.7). We computed 50 times the CIfit and the GBCfit in the regional heterogeneity optimal working point and compared with the homogeneous model (scale = 0; bias = 0) and a spatial null model (generated by shuffling the regions of heterogeneity, preserving the spatial autocorrelation following Burt et al. [2020]; see the Supporting Information, Figure S1, for a rendering of the map and its surrogates). The boxplots show that the heterogeneous model yields the best fitting performance compared with the other two models in the CIfit and GBCfit (*P* < 0.001, Wilcoxon rank sum test with Bonferroni correction).

### Organization of the Heterogeneous Bifurcation Parameters

**Figure 4.**
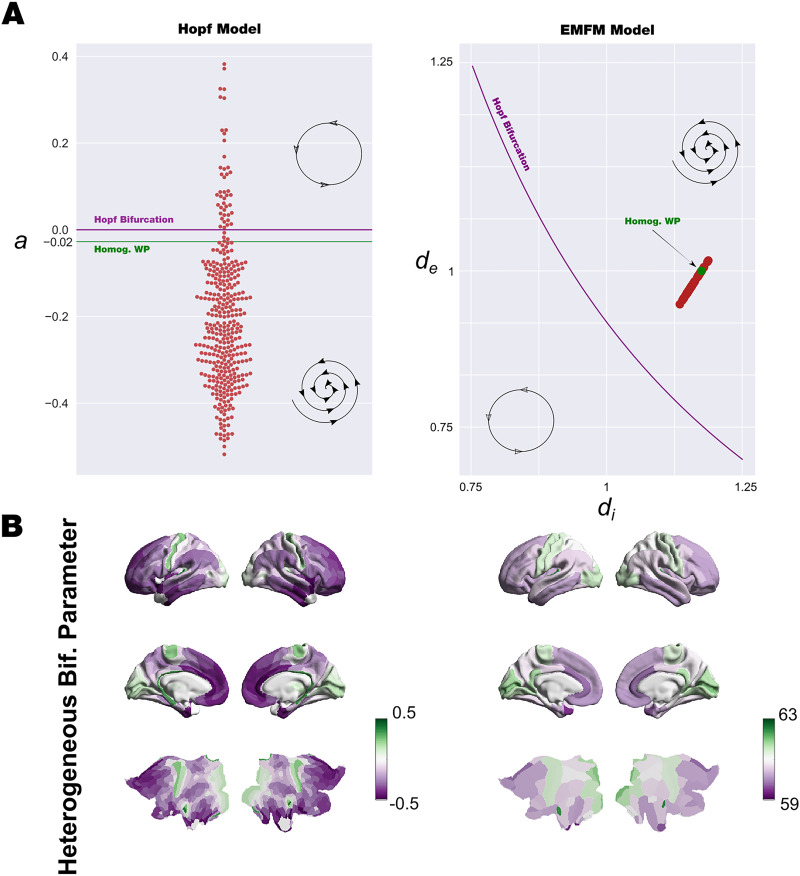
Heterogeneous bifurcation parameters. (A) We obtained the bifurcation parameters for both models shaped by the scaling and bias obtained from the heterogenous fitting optimization procedure described in previous figures. The left panel shows the Hopf whole-brain model organization of the heterogenous bifurcation parameter: a value for each node (red circles), with respect to the Hopf bifurcation point, *a* = 0 (purple line) and the homogenous value, *a* = −0.02 (green line). The right panel shows the exact mean-field whole-brain model organization of the bifurcation parameters *d*_*e*_ and *d*_*i*_. Red circles show the values of *d*_*e*_ and *d*_*i*_ for each node, the bifurcation line is indicated in purple, and the homogeneous optimal working point is indicated in green. (B) The values of heterogeneous bifurcation parameters are rendered onto a brain cortex. The left panel shows the bifurcation parameter *a* of the Hopf whole-brain model, and the right panel shows the norm of the vector defined by each (*d*_*e*_; *d*_*i*_) pair. The same brain areas in both models are shifting toward high values and low values; nevertheless, the distribution with respect to the bifurcation point shows different behavior.

We investigated how the heterogeneous bifurcation parameters are organized in the parameter space with respect to the bifurcation point. We computed the bifurcation parameters for both models shaped by the scaling and bias obtained from the heterogenous fitting optimization procedure described in previous section. We found that the heterogenous bifurcation parameters, *a*_*i*_, in the SL model are mostly located below the Hopf bifurcation point (Figure 4A, left panel). We also observed that the heterogenous bifurcation parameters, *d*_*e*_ and *d*_*i*_, in the exact mean-field model are located below the bifurcation curve (Figure 4A, right panel). Interestingly, in both models the heterogenous bifurcation parameters are distributed around the homogeneous optimal working point (green line/point in SL model and exact mean-field model, Figure 4A).

We rendered the values of the heterogenous bifurcation parameters for both models onto a brain cortex. The bifurcation parameter, *a*, of the SL whole-brain model and the norm of the vector defined by each (*d*_*e*_; *d*_*i*_) pair are shown in the left and right panels of Figure 4B. Remarkably, despite the same brain areas in both models shifting toward high values and low values by construction, the distribution with respect to the bifurcation point shows different behavior.

### The Impact of Regional Heterogeneity in the SL Whole-Brain Model Fitted to Neurodegeneration

**Figure 5.**
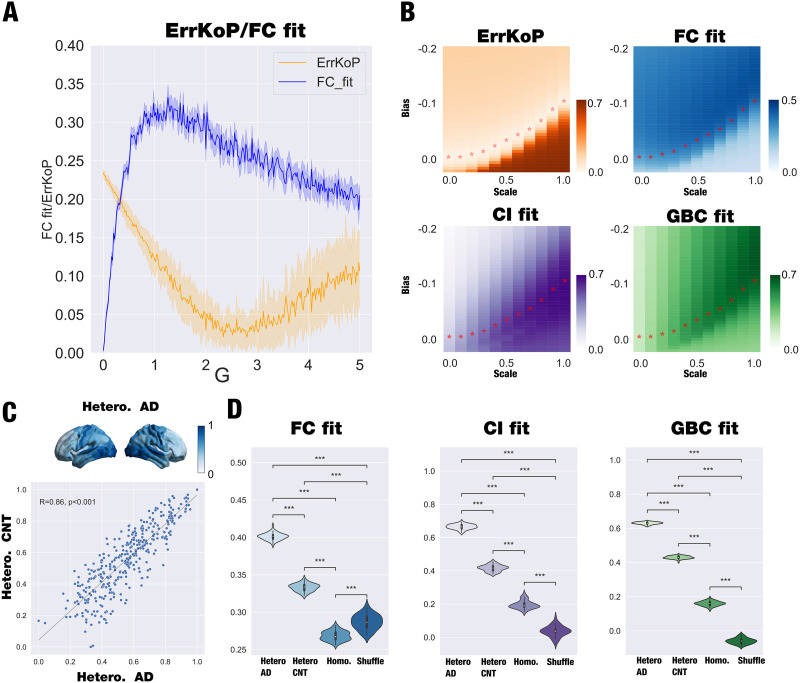
Hopf whole-brain model and the impact of regional functional heterogeneity in Alzheimer’s disease patients. (A) The homogeneous model (*a* = −0.02 for all nodes) was fitted to the empirical data by computing the correlation between model and empirical functional connectivity (FCfit, blue) and absolute error of the model and empirical Kuramoto order parameter (ErrKoP, orange) as a function of the global coupling parameter, *G*. We considered the model’s optimal working point to be the minimum of ErrKoP reached at *G* = 2.8. (B) Using the optimal working point found for the homogeneous model, we introduced regional heterogeneity and assessed its impact by exploring the two-dimensional space determined by the bias and the scaling that directly modify the regional bifurcation parameters. We computed four different fitting measures: the ErrKoP and FCfit; and the global brain connectivity fit (GBC) and the error in a proxy of Granger causality (CIfit), both computed as the Pearson correlation between the empirical and simulated measures. We defined an iso-level curve of ErrKoP, represented by red stars in the four matrices, computed as the value of bias that reaches the minimum value of ErrKoP for each value of scaling. (C) We rendered onto a brain the AD-specific regional heterogeneity (upper panel), and we then presented the high correlation between regional functional heterogeneities of patients and controls (lower panel). (D) We selected the scale value where the maximal CIfit is reached and computed 50 times the CIfit, FCfit, and the GBCfit with the functional AD-specific disease heterogeneity, healthy functional specific heterogeneity, the homogeneous model, and a spatial null model (generated by shuffling the regions of heterogeneity, preserving the autocorrelation). The violins show the comparison of the four models in the three measures, presenting statistical significance for all the cases (*** *P* < 0.001, Wilcoxon rank sum test with Bonferroni correction).

We moved further and investigated the role of disease-specific regional heterogeneities to model Alzheimer’s disease (AD) patients. To do so, we assessed the homogeneous SL whole-brain model performance in reproducing empirical properties of resting-state data of Alzheimer’s in the fine parcellation with 360 brain regions (Glasser et al., 2016). We explored the global coupling parameter *G* with all regional bifurcation parameters *a*_*i*_ = −0.02. Figure 5A shows how well the model fits as a function of *G* in terms of FCfit and ErrKoP, and we defined the homogeneous model optimal working point as the minimum of ErrKoP, which shows a clear optimum at *G* = 2.8.

We then studied how disease-specific regional functional heterogeneity given by the node level of brain connectivity (GBC, see Methods) affects the fitting of the empirical properties. We introduced this heterogeneity as in the previous sections and computed the level of fitting of the FC, GBC, KoP, and a slightly different version of CI due to the sampling rate of patients’ data (see Methods). We exhaustively explored the bias and scale of the heterogeneous model, and in Figure 5B we show the evolution of the fitting of the four measures. As in previous sections, we identified an iso-level curve of ErrKoP, and we identified the optimal working point of the heterogeneous model as the maximum CIfit within the iso-level curve of ErrKoP.

Finally, we compared the performance of the heterogeneous model in fitting the empirical observables with the homogeneous model and the null hypothesis provided by the heterogeneity spatial shuffling (as in the previous sections). We also assessed the impact of including the functional heterogeneity given by the GBC of healthy controls (CNT) in the model-fitting performance of Alzheimer’s disease patients’ data. In Figure 5C, upper panel, we rendered onto a brain the AD-specific regional heterogeneity; in the lower panel, we showed the high correlation between regional functional heterogeneities of patients and controls. Figure 5D shows the statistical difference between the AD-specific heterogeneous model, the CNT-specific heterogeneous model, the homogeneous model, and the spatial shuffling (generated by shuffling the regions of heterogeneity, preserving the spatial autocorrelation following Burt et al. [2020]; see the Supporting Information, Figure S1, for a rendering of the map and its surrogates) for 50 runs of each model (*P* < 0.001, Wilcoxon rank sum test with Bonferroni correction). We extended these results to a different fitting metric, such as the Euclidian distance between FC matrices, to investigate to what extent these models can be useful to a different measure (see the Supporting Information, Figure S3). We noted that slight variations in the heterogeneity, such as those presented between the controls and disease functional maps (highly correlated, see Figure 5C), yield significantly different fitting levels. This high sensitivity could be related to the relationship between maps and the underlying network that couples the system. We computed the differences between the two sources of heterogeneity (GBC_CNT_ − GBC_AD_) and compared them with the weighted degree of the underlying structural connectivity (Supporting Information, Figure S4). We found that both measures are correlated (*R* = 0.31, *p* < 0.001), showing that the regions where healthy controls present more GBC than AD patients correspond to highly connected regions. Thus, the differences in these regions could strongly impact the whole-brain dynamics, providing a possible explanation of the difference in the level of fitting between both heterogeneities. In summary, we demonstrated that the best model-fitting capability is reached when we include the corresponding disease-specific functional heterogeneity.

## DISCUSSION

In this study, we extended previous work focused on investigating the dynamical consequences of local heterogeneities in asynchronous brain activity to the case of synchronous oscillatory behavior. We implemented phenomenological Stuart–Landau and exact mean-field whole-brain models of fMRI dynamics informed by T1w/T2w regional heterogeneity to evaluate the dynamical consequences of these variations in the oscillatory regime. The fit of the two models that are able to present oscillatory behavior with different levels of abstraction allows us to widely explore the static and dynamic implication of the inclusion of regional heterogeneities for modeling a large-scale dataset of resting-state fMRI recordings. We then identified that disease-specific regional functional heterogeneity also brings dynamical consequences within the oscillatory regime in fMRI recordings from patients with neurodegeneration (Alzheimer’s disease). In the following paragraphs, we discuss our findings in light of whole-brain modeling and regional heterogeneity within an oscillatory context, extending the findings of previous work in asynchronous models.

It has been demonstrated that cortical areas present multiple sources of regional variations, such as myeloarchitecture, cytoarchitecture, the density of neurotransmitter receptors, gene expressions, excitation/inhibition ratio, and interregional connectivity, among others (Deco et al., 2018; Deco, Kringelbach, et al., 2021; Deco et al., 2014; Huntenburg et al., 2018; Margulies et al., 2016; X. J. Wang, 2020). The relation between the spatial structure variation and the function has largely been studied, and the specific dynamical consequences of each of these heterogeneities have been investigated through whole-brain models. In previous work, Chaudhuri et al. (2015) included a hierarchical ordering of macaque cortical areas based on linearly scaling the excitatory input strength of each population into a mean-field model, changing the dynamical scenario from a single stable point toward a bistable state accordingly. In the same direction, Demirtaş et al. (2019) and Deco, Kringelbach, et al. (2021) included different sources of heterogeneity (comprising the T1w/T2w) to modulate the gain of the local neuronal response functions of the corresponding excitatory and inhibitory pools of each brain region. So far, these approaches have considered biophysical mean-field asynchronous models. Here, first, we introduced heterogeneity in the phenomenological SL whole-brain model by modifying the local bifurcation parameter of each region by the inclusion of a bias and a scaling that modulates the regional heterogeneity given by the T1w/T2w. In this sense, the heterogeneity can change the dynamical scenario for a single region from oscillatory toward noise dynamics, or vice versa. The relationship between the bifurcation parameter and the local excitation/inhibition ratio can be inferred by studying the bifurcation diagram of more biophysically realistic models, such as the Wilson–Cowan model (Cowan et al., 2016). In line with previous results on mean-field non-oscillatory models, we found that the inclusion of T1w/T2w regional heterogeneity has substantial implications on fitting static measures, such as the functional connectivity (FC) and the global brain connectivity (GBC) (Deco, Kringelbach, et al., 2021; Demirtaş et al., 2019). We also found that this heterogeneity has implications in the information transmission flow measured by the causal ignition within the NDTE framework (Deco, Vidaurre, et al., 2021). Specifically, this measure indicates the influence that each brain region presents over the whole level of brain synchronization.

We further investigated the implications that regional heterogeneities have on modeling brain activity by building a biophysically grounded mean-field model capable of reproducing the switch between oscillations and stable fixed points. In the last few years, several authors have modeled different observables from empirical fMRI data using Stuart–Landau whole-brain models (Deco & Kringelbach, 2020; Ghosh et al., 2008; Ipiña et al., 2020; Jobst et al., 2017). On the other hand, more sophisticated biophysical grounded models, such as mean-field models, were also successfully used to build whole-brain models capable of modeling empirical data from fMRI recordings (Breakspear et al., 2003; Deco & Jirsa, 2012; Deco, Kringelbach, et al., 2017; Deco et al., 2014; di Volo et al., 2019; Freyer et al., 2012; Ghosh et al., 2008; Herzog et al., 2020; Honey et al., 2007; Ponce-Alvarez et al., 2015). However, what level of abstraction is considered in the local dynamics to construct whole-brain models is an open question and focus of ongoing research. We hypothesize that one reason why the SL whole-brain model is one of the best models that fit to fMRI BOLD imaging despite its simplicity (Deco, Kringelbach, et al., 2017) is the capability of the Stuart–Landau model to capture the oscillatory nature of brain signals. In contrast, the spiking and standard mean field are asynchronous and unable to capture these oscillatory couplings. Furthermore, when these models are expressly set on the edge of the bifurcation point, they have demonstrated an excellent capability to reproduce metastability (Deco, Kringelbach, et al., 2017) and critical behavior of brain activity (Chialvo, 2010; Cocchi et al., 2017; Perl et al., 2021). In order to shed light on this question, we included oscillations in the mean-field model and compared them with the SL whole-brain model results.

We implemented an exact mean-field whole-brain model based on the mean-field description for populations of QIF neurons derived in Montbrió et al. (2015). We considered the local dynamics of each brain region to be described by a pool of inhibitory and excitatory neurons, coupled via structural connectivity with the other brain regions. We found that, as in the SL model, the best fit for this mean-field model is slightly below the Hopf bifurcation. We were able to fit the functional connectivity and the level of synchronization as well as other homogeneous whole-brain models based on mean-field local dynamics (Deco et al., 2018; Deco, Kringelbach, et al., 2021; Demirtaş et al., 2019; P. Wang et al., 2019). We investigated the implication of the regional heterogeneities, as in the SL model, when we model empirical observables by equally modulating the half-wide distribution of the variability within each population of inhibitory and excitatory neurons. We found that the T1w/T2w heterogeneity plays a crucial role in improving the level of fitting of GBC and causal ignition using this biophysical mean-field model capable of oscillatory dynamics.

We evaluated how the heterogeneous bifurcation parameters, that is, the ones that reach the best heterogenous level of fitting, were distributed with respect to the Hopf bifurcation. In both scenarios, the values are distributed around the bifurcation point, providing an indication that the optimal dynamical behavior is on the edge of criticality, between fluctuations and oscillations, as suggested by whole-brain models in previous research (Deco et al., 2013; Deco, Kringelbach, et al., 2017; Spiegler et al., 2016) and demonstrated in more detail in Freyer et al. (2012).

Finally, we investigated the dynamical consequences by including disease-specific regional heterogeneities based on functional fMRI recordings of Alzheimer’s disease patients. In general, neurodegenerative diseases such AD and bvFTD (behavioral variant frontotemporal dementia) are characterized by heterogeneity across different brain levels involving both structure and dynamics (Maestú et al., 2021; Mehta et al., 2013; Verdi et al., 2021), challenging the assumptions of spatially homogeneous models. Specifically, different measures on patients such as positron emission tomography (PET), which informs the accumulation of pathological proteins in the brain, such as amyloid-beta plaques (Nordberg, 2004) and neurofibrillary tau tangles (Schöll et al., 2016), or atrophy maps from fRMI, which may constitute signs of neurodegeneration (Schoonenboom et al., 2004), provide different sources of heterogeneity. However, insufficient mechanistic accounts of neurodegeneration prevent the development of integrative models of neurodegenerative diseases. We proposed investigating the heterogeneity implications in modeling AD data by following the approach proposed by Kong et al. (2021), who demonstrated that functional gradients improve the model-fitting capabilities of static and dynamic empirical observables from resting-state fMRI. We defined the functional heterogeneity as the GBC, which stands for how connected each brain region is with the rest of the brain in terms of functionality, and we computed for AD and healthy controls. Based on the results presented here and previous work, which successfully fit AD data with the SL model (Demirtaş et al., 2017), we used the SL model because it is less computationally intensive than the exact mean-field model and reaches a similar fitting level. We found that the AD functional heterogeneity in the model yielded a more accurate reproduction of the spatiotemporal structure of empirical FC, GBC, and CI. Note that this model largely overperformed the homogeneous model. Similar results were found by Kong et al. (2021), including functional gradient as regional heterogeneity in a mean-field model fitting the static and dynamic FC. We compared fitting improvements by including healthy control functional heterogeneity to fit the empirical observables for AD. Notably, despite that the two specific regional heterogeneities, healthy and AD, are highly correlated (*r* = 0.86), we found that the model with healthy control heterogeneity overperforms the homogeneous case but is not as good as the specific AD regional heterogeneity. We hypothesize that this fitting difference could be related to the fact that regions with the highest differences in heterogeneity between CNT and AD are the most connected by the underlying structure in the brain. Our results on AD fMRI data are proof of concept in fulfilling the gap of modeling neurodegeneration, providing mechanisms to include regional heterogeneity to build integrative models that allow the integration of different recording modalities.

In summary, we showed that the best fits are obtained by including the disease-specific functional heterogeneity from the AD patients, with poor results obtained when we used the GBC from healthy controls or the homogeneous model. This result prompts the need to discuss the relationship between this functional map and local dynamics. AD is linked to altered cellular energy metabolism (Gu et al., 2012), excitation/inhibition ratio (Lopatina et al., 2019; Maestú et al., 2021; Mehta et al., 2013), and neurotrophic factor release (Murer et al., 2001), impairing neural microcircuit function (Palop & Mucke, 2016). In particular, the excitation/inhibition ratio seems to play an important role in characterizing AD (Benussi et al., 2020; Cammisuli et al., 2022; Maestú et al., 2021). Considering that the Hopf bifurcation parameter and the local excitation/inhibition ratio can be related (which can be inferred by studying the bifurcation diagram of the Wilson–Cowan model [Cowan et al., 2016]), it is likely that this feature of cortical dynamics is being captured by the specific disease functional heterogeneity that directly modulates the local bifurcation parameter. Nevertheless, we acknowledge that with imaging technologies the number of high-resolution reference maps of brain structure and function are increasing (e.g., frequency gradients maps [Mahjoory et al., 2020] or the gene expression and protein density as used by Hansen et al. [2022]). In this direction, the comparison of our disease functional maps and these reference maps are not investigated here because it is out of the scope of this work. We focused on proposing a dynamic mechanism of the impact of including a map in oscillatory models, but extension to the evaluation of different maps will be considered in further studies. In particular, we propose leveraging the work of Markello et al. (2022), who developed an excellent tool to interpret structural and functional brain maps.

Overall, we demonstrated that two conceptually different whole-brain models presenting similar dynamic scenarios are equally good at fitting static and dynamic empirical data from resting-state fMRI recordings of healthy participants. Notably, the optimal working point of both models is at the edge of the Hopf bifurcation. We then showed that the dynamical consequences of the regional heterogeneities in the fitting capacity are similar in both models. To the extent that the SL whole-brain model performs similarly to an exact mean-field biophysically grounded whole-brain model, we conclude that the SL whole-brain model represents a suitable level of abstraction that captures one of the main dynamic behaviors of brain signals. Finally, we showed that functional specific-disease regional heterogeneities in the SL whole-brain model are relevant to improving the model fitting of empirical observables from fMRI recordings from participants with Alzheimer’s disease. We demonstrated that models with structural (Tw1/Tw2 ratio) or functional regional heterogeneities perform better than the homogeneous model. Furthermore, we showed that the specificity of the heterogeneity has a positive impact on the model’s performance; that is, the model performs better when the inclusion of the heterogeneity is related to the condition that has been modeled. A further direction is to include at the same time different classes of heterogeneities, structural and functional, specific and nonspecific, to improve model-fitting capacities but also to establish hierarchies between different heterogeneities. Steps in that direction were given by Kong et al. (2021) for healthy resting-state participants. We postulated that the combination of functional and structural sources of local heterogeneities could provide better model performance, and this framework can provide an analytical tool to determine the relevance of each class of heterogeneity.

## SUPPORTING INFORMATION

Supporting information for this article is available at https://doi.org/10.1162/netn_a_00299.

## AUTHOR CONTRIBUTIONS

Yonatan Sanz Perl: Conceptualization; Formal analysis; Investigation; Methodology; Software; Writing – original draft; Writing – review & editing. Gorka Zamora-Lopez: Investigation; Methodology; Writing – review & editing. Ernest Montbrió: Formal analysis; Investigation; Methodology; Writing – review & editing. Martí Monge-Asensio: Formal analysis; Visualization; Writing – review & editing. Jakub Vohryzek: Methodology; Writing – review & editing. Sol Fittipaldi: Data curation; Writing – review & editing. Cecilia González Campo: Data curation; Writing – review & editing. Sebastián Moguilner: Data curation; Writing – review & editing. Agustín Ibañez: Data curation; Writing – review & editing. Enzo Tagliazucchi: Formal analysis; Methodology; Writing – review & editing. B. T. Thomas Yeo: Methodology; Writing – review & editing. Morten Kringelbach: Conceptualization; Methodology; Software; Writing – review & editing. Gustavo Deco: Conceptualization; Formal analysis; Investigation; Methodology; Software; Supervision; Writing – original draft; Writing – review & editing.

## FUNDING INFORMATION

Yonatan Sanz Perl, H2020 Marie Skłodowska-Curie Actions (https://dx.doi.org/10.13039/100010665), Award ID: 896354. Yonatan Sanz Perl, EU H2020 FET Flagship program and by European Union’s Horizon 2020, Award ID: 945539. Gorka Zamora-Lopez, EU H2020 FET Flagship program and by European Union’s Horizon 2020, Award ID: 945539. Ernest Montbrió, Ministerio de Educación y Formación Profesional (https://dx.doi.org/10.13039/501100020636), Award ID: PID2019-109918GB-I00/AEI/10.13039/501100011033. Agustín Ibañez, CONICET, Award ID: FONCYT- PICT (2017-1818, 2017-1820). Agustín Ibañez, Takeda, Award ID: CW2680521. Agustín Ibañez, Alzheimer’s Association, Award ID: SG-20-725707-ReDLat. Agustín Ibañez, National Institutes of Aging of the National Institutes of Health, Award ID: R01AG057234. Agustín Ibañez, ANID/FONDECYT Regular, Award ID: 1210195, 1210176, 1220995. Agustín Ibañez, ANID/FONDAP, Award ID: 15150012. Agustín Ibañez, ANID/PIA/ANILLOS, Award ID: ACT210096. Enzo Tagliazucchi, CONICET, Award ID: PICT-2019-02294. Enzo Tagliazucchi, ANID/FONDECYT, Award ID: 1220995. Morten Kringelbach, Danish National Research Foundation, Award ID: DNRF117. Gustavo Deco, EU H2020 FET Flagship program and by European Union’s Horizon 2020, Award ID: 945539. Gustavo Deco, Spanish Ministry of Science, Innovation, and Universities (MCIU), Award ID: ref. PID2019-105772GB-I00 MCIU AEI.

## TECHNICAL TERMS

Hopf bifurcation: The critical point where the system changes the solutions from fixed point to a self-sustained oscillatory behavior.
Global brain connectivity (GBC): This measure stands for the mean level of functional connectivity that each brain region has with the rest of brain regions.
Functional connectivity (FC): The pairwise Pearson’s correlation between fMRI time series.
Kuramoto order parameter (KoP): This measure reflects the level of global synchronization of a system of *N* oscillators; when KoP = 1, the system is fully synchronized.
Causal ignition (CI): This measure reflects the direct causality between the activity of one region and the global level of synchronization of the system.
Hopf whole-brain model: Computational model describing the whole-brain dynamics, where the dynamics of each brain region is described by a Stuart-Landau oscillator.
Exact mean-field whole-brain model: Model where the dynamics of each brain region are described by the mean-field equations exactly derived from a large population of all-to-all coupled quadratic integrate-and-fire (QIF) neurons.
