# Supplementary material for: The impact of regional heterogeneity in whole-brain dynamics in the presence of oscillations
Source: Netw Neurosci. 2023 Jun 30;7(2):632–60. doi: 10.1162/netn_a_00299 (PMC10312285; doi:10.1162/netn_a_00299)
Supplement: Supplementary file 1 [file netn-7-2-632-s001.pdf]

Perl, Y. S., Zamora-Lopez, G., Montbrió, E., Monge-Asensio, M., Vohryzek, J., Fittipaldi, S., Campo, C. G., Moguilner, S., Ibañez, A., Tagliazucchi, E., Yeo, B. T. T., Kringelbach, M. L. & Deco, G. (2022). Supporting information for "The impact of regional heterogeneity in whole-brain dynamics in the presence of oscillations." *Network Neuroscience*. Advance publication. https://doi.org/10.1162/netn_a_00299

Supplementary Information: "The impact of regional heterogeneity in whole-brain dynamics in the presence of oscillations"

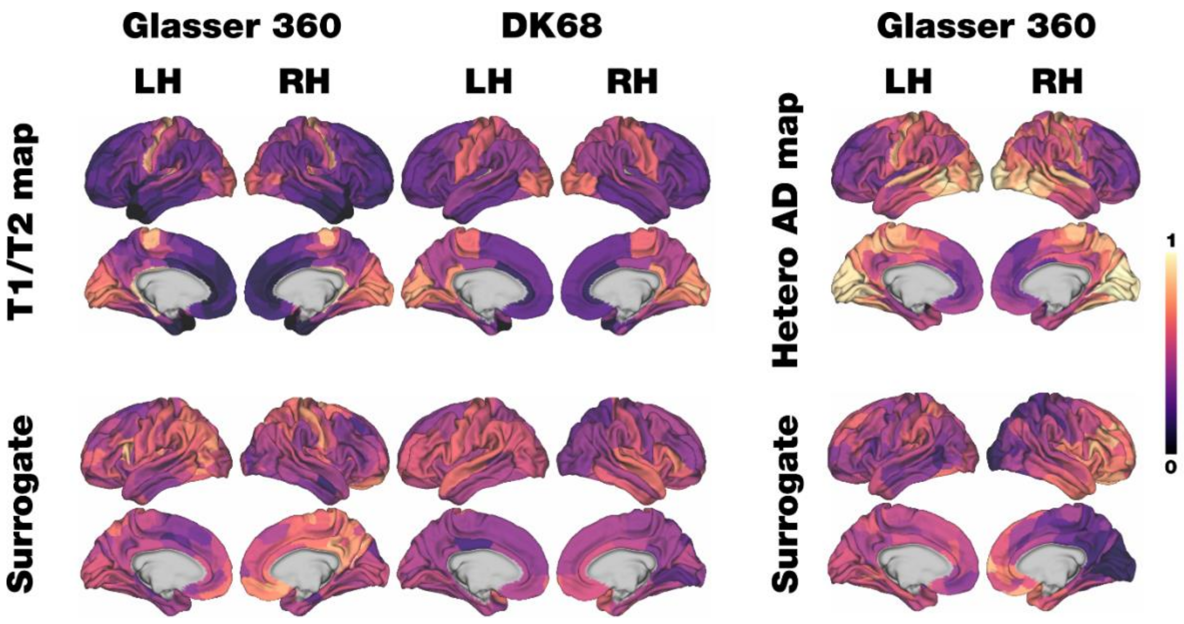

***Figure S1**: Brain renders of the original heterogeneity maps used in the work (first row) and the surrogated generated preserving the spatial autocorrelation as is proposed in* (Burt et al., 2020) *(lower row). The first and second column show the T1/T2 ratio for both parcellations Glasser 360 and Desikan-Killiany 68 (corresponding to Fig 2 and Fig 3 of main text, respectively). The rightmost column shows the specific-disease functional heterogeneity for AD in Glasser 360 parcellation (upper) and its surrogate (bottom).*

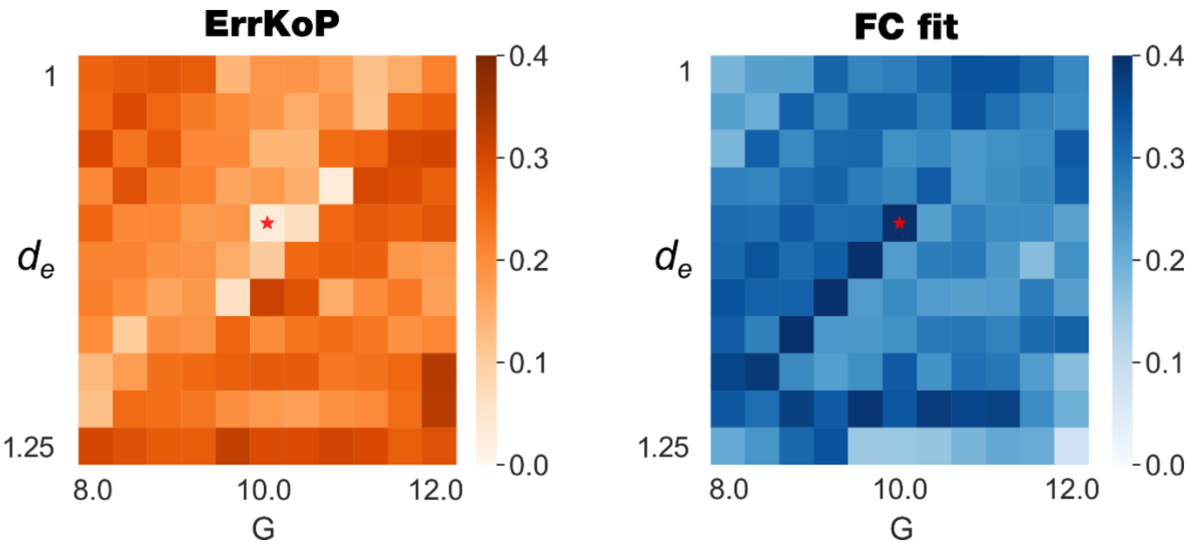

***Figure S2**: The homogenous model fitted in the parameter space determined by G and d_e. The homogeneous model (η=40/30;d_i=1; J_{ee}=50/√40; J_{ie}=20/√40;J_{ie}=J_{ii}=-20/√40 , equally for all nodes) was fitted to the empirical data by computing the correlation between model and empirical functional connectivity (FCfit, blue colors) and absolute error of the model and empirical Kuramoto order Parameter (ErrKoP, orange colors) as a function of the global coupling parameter, G and the rescaled dispersion of the local level of heterogeneity of inhibitory population, de. We found a similar structure and similar fitting values compared with the original exploration presented in Fig 3.*

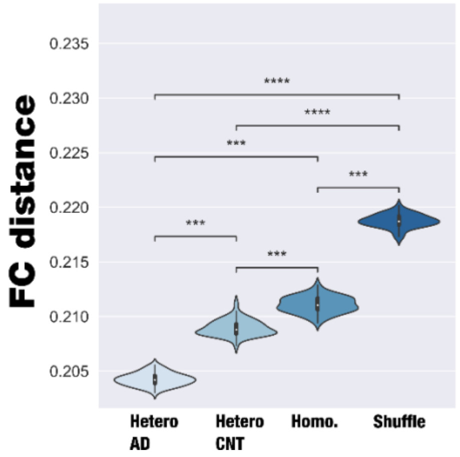

Figure S3: *Whole-brain model of coupled Stuart-Landau oscillators show consequences of including regional heterogeneity in terms of the fitting to empirical FC quantified through the Euclidian distance between FC matrices. We selected the scale value where the maximal CIfit is reached and computed 50 times the FC distance with the functional AD-specific disease heterogeneity, healthy functional specific heterogeneity, the homogeneous model and a spatial null model (generated by shuffling the regions of heterogeneity preserving the spatial autocorrelation). The violins show the comparison of the four models in the three measures presenting statistical significance for all the cases (*** stand for P<0.001, Wilcoxon rank sum test with Bonferoni correction).*

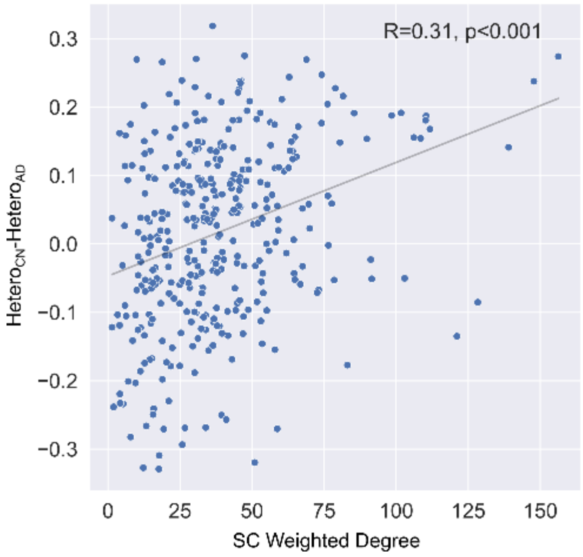

Figure S4: *The difference between functional heterogeneity from healthy controls and Alzheimer patients as a function of the weighted degree of the underlying structural connectivity. The highly significant correlation between both variables is 0.31, and in particular the highest difference between both heterogeneities corresponds with higher degree in the underlying connectivity. This relation could explain the differences in the level of fitting when the heterogeneity is the GBC from AD or the GBC from CN.*
